# Novel compound heterozygous mutations in *TELO2* in a patient with severe expression of You‐Hoover‐Fong syndrome

**DOI:** 10.1002/mgg3.287

**Published:** 2017-07-28

**Authors:** Shahida Moosa, Janine Altmüller, Troels Lyngbye, Rikke Christensen, Yun Li, Peter Nürnberg, Gökhan Yigit, Ida Vogel, Bernd Wollnik

**Affiliations:** ^1^ Institute of Human Genetics University Medical Center Göttingen Göttingen Germany; ^2^ Institute of Human Genetics University of Cologne Cologne Germany; ^3^ Cologne Center for Genomics University of Cologne Cologne Germany; ^4^ The Centre for Deafblindness and Hearing Loss Aalborg Denmark; ^5^ Department of Clinical Genetics Aarhus University Hospital Aarhus Denmark

**Keywords:** Novel mutations, syndromic ID, TELO2, You‐Hoover‐Fong syndrome

## Abstract

**Background:**

Very recently, compound heterozygous loss‐of‐function mutations in *TELO2* were shown to underlie the newly‐described You‐Hoover‐Fong syndrome. TELO2 forms part of the co‐chaperone triple T complex (TTT complex), which plays an important role in the maturation and stabilization of the phosphatidylinositol 3‐kinase‐related protein kinases (PIKKs). Patients with mutations in *TELO2* present with microcephaly and associated intellectual disability, postnatal growth retardation and dysmorphic features. Here, we describe Danish sisters with two novel mutations in *TELO2*. In particular, we highlight the clinical features of the 22‐year index patient, which are more severe than the original patients described, thereby expanding the clinical spectrum of YHFS.

**Methods:**

The index patient was clinically examined and subsequently exome sequencing on her DNA was performed using the NimbleGen SeqCap EZ Human Exome Library v2.0 enrichment kit on an Illumina HiSeq2000 sequencer.

**Results:**

Two novel, compound heterozygous mutations in *TELO2* were identified in the index patient and her deceased older sister. Both have clinical features in keeping with the original YHFS patients, although the index patient seems to represent the severe end of the clinical spectrum with very marked prenatal onset growth retardation and microcephaly, severe global developmental delay and facial dysmorphic features. Additional clinical findings include eye anomalies (bilateral congenital cataracts, retinitis pigmentosa, convergent squint), bilateral conductive hearing loss, an abnormal kidney and seizures.

**Conclusion:**

This report of Danish siblings with YHFS serves to expand the presentation of this new syndrome to include features in keeping with a form of microcephalic primordial dwarfism on the severe end of the clinical spectrum, and adds two novel mutations to the *TELO2* mutational spectrum.

## Introduction

Very recently, compound heterozygous loss‐of‐function mutations in *TELO2* (OMIM 611140) were described in six individuals with a syndromic form of microcephaly and intellectual disability (ID), now eponymously named You‐Hoover‐Fong syndrome (YHFS; OMIM616954) (You et al. 2016). TELO2 forms part of the co‐chaperone triple T complex (TTT complex), along with Tel two‐interacting protein 1 (TTI1; OMIM614425) and Tel two‐interacting protein 2 (TTI2; OMIM614426). Together, this complex plays an important role in the maturation and stabilization of the phosphatidylinositol 3‐kinase‐related protein kinases (PIKKs). Patients with mutations in *TELO2* present with intellectual disability, delayed development, microcephaly, postnatal growth retardation and dysmorphic features, which – at least in part – are common features of several PIKK‐related disorders.

We present a further family with YHFS and include a description of the oldest patient described to date, a 22‐year‐old Danish girl with two novel compound heterozygous mutations in *TELO2*. We highlight her clinical features, which include severe growth retardation and microcephaly of prenatal onset and marked global developmental delay; thus, a more severe presentation than seen in the original *TELO2* mutation‐positive patients. We therefore expand both the clinical presentation and the mutational spectrum of this newly‐described disorder.

## Methods

Using standard protocols, DNA from the index patient and both parents was extracted from peripheral blood lymphocytes, while DNA from the older deceased sister was extracted from a bloodspot from her newborn screening Guthrie card. Whole exome sequencing (WES) was performed on DNA from the index patient. The study was approved by the ethics committee of the University Medical Center Göttingen and informed consent was obtained from the family for the molecular testing and publication of results and patient photographs. The exome data were analyzed by the MM‐Team, the multidisciplinary NGS analysis team of the Institute of Human Genetics, University Medical Center Göttingen, comprising medical doctors, biologists, biochemists and bioinformaticians. According to the MM‐Team analysis pipeline, each dataset is independently analyzed by four members. Likely‐causative variants are further subjected to literature searches and in silico pathogenicity analyses. These are then discussed considering the presenting phenotype and potential causative variants are confirmed by Sanger sequencing, followed by family segregation studies. A final decision regarding the variants is made by consensus in the MM‐team, based on the resultant evidence for pathogenicity. WES data were analyzed based on a presumed autosomal recessive inheritance pattern (two affected siblings), filtering for rare variants. Additionally, the data were interrogated for variants in known genes for primordial dwarfism and microcephaly, due to the clinical differential diagnosis.

## Results

### Clinical report

**Figure 1:**
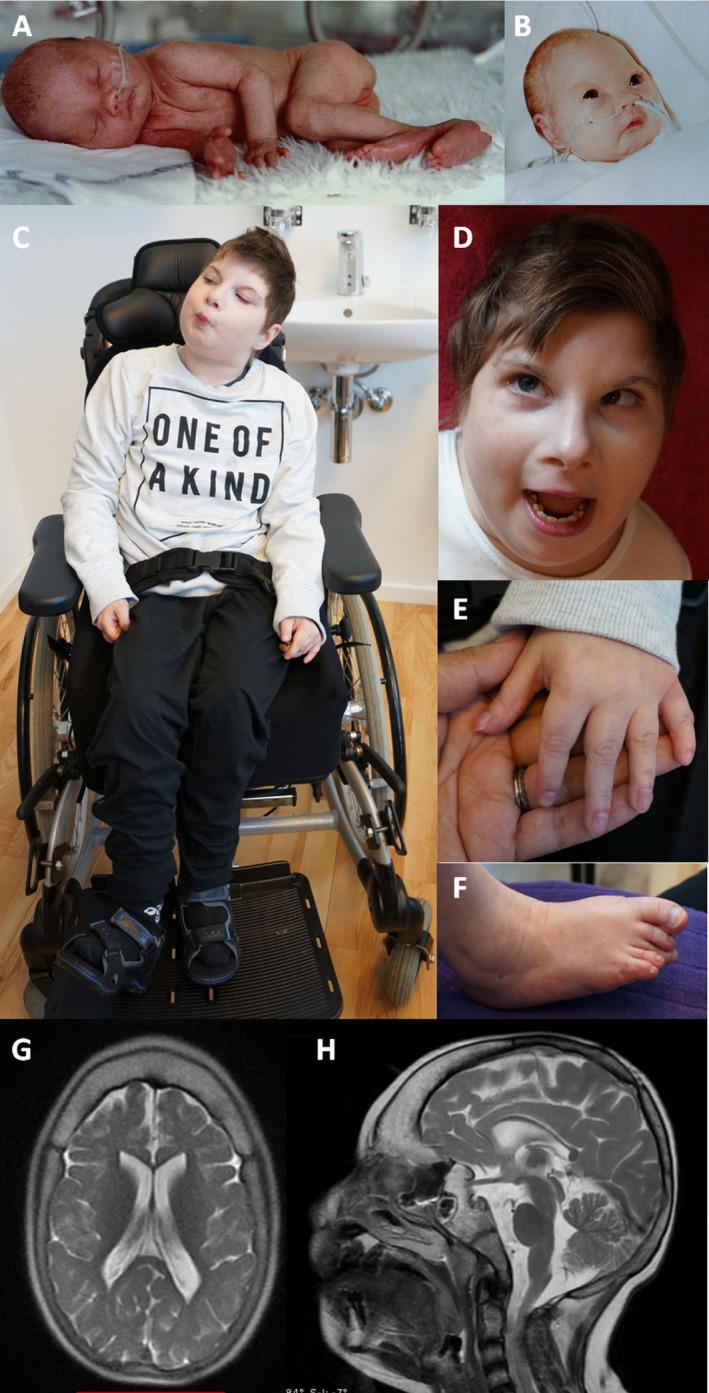
Clinical photographs showing dysmorphic features of the two siblings. (A) and (B) Older sister as a newborn: microcephaly, hypertelorism, upslanted palpebral fissures, flat nasal bridge, downturned corners of the mouth, micrognathia, fifth finger clinodactyly, rocker‐bottom feet. Index patient aged 22 years (C) Full body image (D) Craniofacial features: microcephaly, convergent squint, hypoplastic alae nasi, micrognathia (E) Small hands with tapering fingers and fifth digit clinodactyly (F) Right foot showing overlapping toes and hypoplastic toe nails (G) and (H) MRI brain (index patient): Axial and sagittal sections showing microcephaly and thickening of frontal and sphenoid bones skull. Cortical patterning and intracranial structures appear normal.

The index patient is a currently 22‐year‐old female who is the third daughter of nonconsanguineous, healthy Danish parents. Her eldest sister is healthy. The second daughter was born at 35 weeks’ gestation with a birth weight of 1645 kg (<3rd centile) and a length of 42 cm (3rd centile). She was admitted immediately after birth due to jaundice and the suspicion of an underlying genetic disorder. She had facial dysmorphic features (hypertelorism, upslanted palpebral fissures, downturned corners of the mouth, micro‐retrognathia), a cleft palate, bilateral single palmar creases, bilateral clinodactyly of the fifth fingers with nail hypoplasia, contractures at the hips and knees and rocker‐bottom feet (Fig. 1A,B). The karyotype was normal (46,XX). She had a loud systolic murmur, was treated for jaundice, and developed seizures, apneas and several episodes of bradycardia. She passed away in hospital after a respiratory infection at the age of 2 months. Her head circumference at 2 months was 30 cm (−8,5 SD – equivalent of the mean for 32 weeks’ gestation).

The index patient presented with prenatal onset microcephaly, growth retardation, severe psychomotor developmental delay and intellectual disability and dysmorphic features (Fig. 1C–F). At birth, she weighed 1595 kg (<3rd centile) and her head circumference was 28 cm (<3rd centile) (plotted at 36 weeks’ gestation). Further investigation revealed that she had bilateral congenital cataracts, an abnormal kidney with a double outlet, an annular pancreas and contractures at the hips and knees. Currently, she has bilateral conductive hearing loss, a convergent squint, retinitis pigmentosa, subluxed hips and is generally hypertonic and spastic. She has not learned to sit independently, has no words, and responds mainly to tactile stimuli. She developed seizures at age 12. At 22 years, her head measures 39 cm (−10,7 SD – equivalent of the mean for 3 months old), her length approximately 120 cm (−6,9 SD – equivalent of the mean for 6 years old) and her weight 19 kg (<−3 SD – equivalent of the mean for 5 years old). She has very small hands with tapering fingers and fifth digit clinodactyly and small feet with overlapping toes and hypoplastic toe nails. Her cardiac examination was normal and an MRI of her brain showed no major structural abnormalities (Fig. 1G,H). Karyotype and subsequent array CGH were also normal. Due to her significant prenatal onset microcephaly and short stature (both <4SD), a form of microcephalic primordial dwarfism was initially suspected.

### Mutations identified

The analysis of the WES data initially focused on all genes known to cause primordial dwarfism and microcephaly. These genes were adequately covered and no variants were identified in any of them. Filtering for rare, highly damaging variants yielded just five heterozygous frameshift variants. Four of these were excluded as they either had not been associated with a clinical phenotype to date, or in one case, caused an unrelated phenotype in the homozygous or compound heterozygous state. There were no further variants in these four genes, which could have resulted in compound heterozygosity. *TELO2* (RefSeq transcript NM_016111.3) was the only gene in which compound heterozygous variants were identified (combination of a frameshift and missense). Bi‐allelic mutations in *TELO2* were recently described in patients with an overlapping phenotype; thus, we regarded *TELO2* as an excellent candidate to explain the clinical phenotype in our patients.

**Figure 2:**
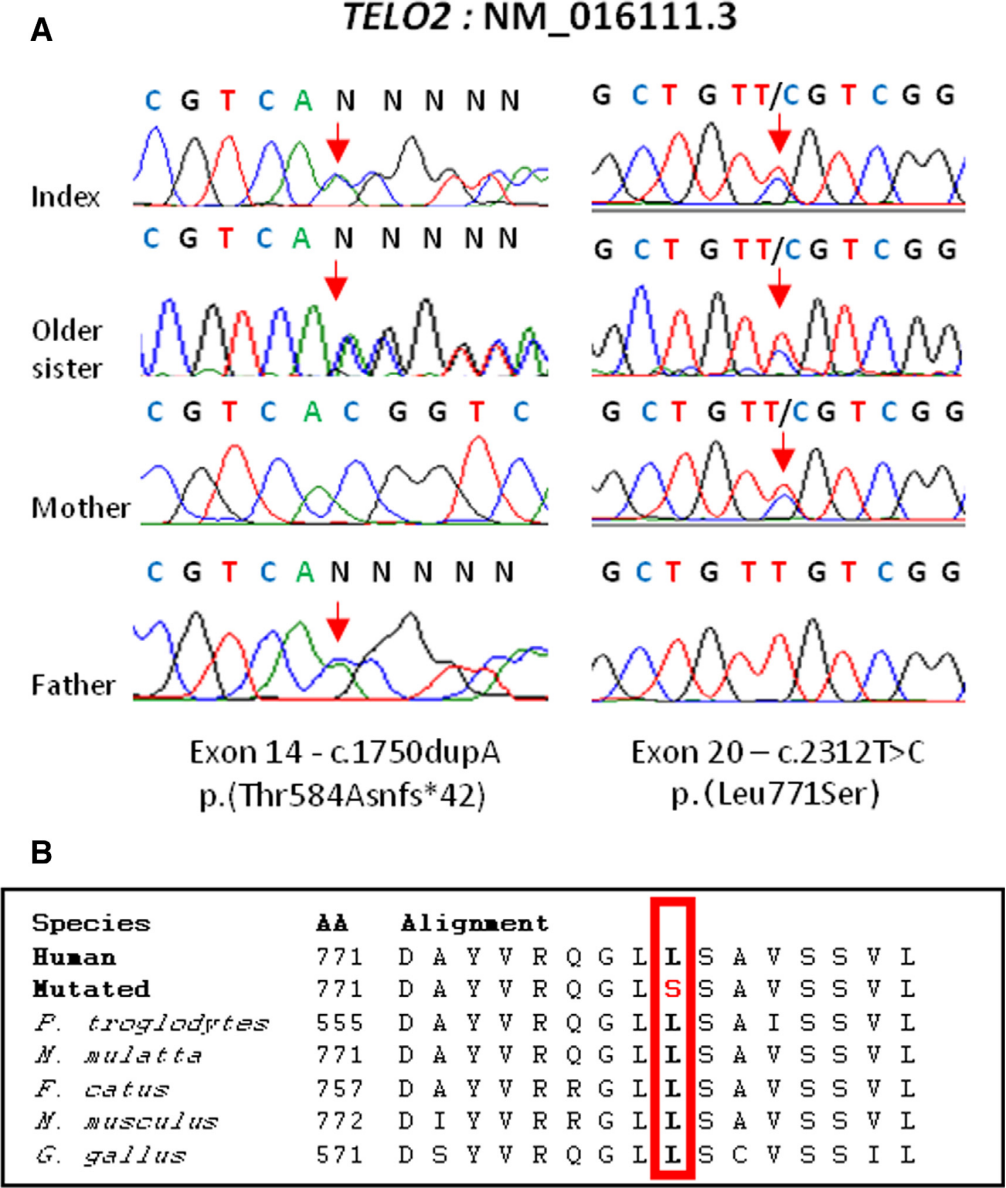
Mutations identified. (A) Chromatograms showing the mutations identified in Exons 14 and 20, respectively of *TELO2*. Both siblings are compound heterozygous, while the parents are heterozygous carriers of one mutation each (B) Cross‐species alignment showing conservation of the p.(Leu771Ser) residue (boxed in red), affected by the missense mutation c.2312T>C.

The frameshift variant in *TELO2*, located in exon 14, c.1750dupA, is predicted to lead to premature termination of the protein, p.(Thr584Asnfs*42). It is absent from all population variation databases (dbSNP, EVS, ExAC, 1000Genomes). The second *TELO2* variant, c.2312T>C, is located in exon 20. At the protein level, this variant is predicted to substitute a highly conserved leucine by serine at amino acid position 771, p.(Leu771Ser) (Fig. 2B). This variant is present at a very low frequency in the ExAC (3 of 72300 alleles) and ESP (1 of 4393 alleles) databases and is not described in homozygosity. As previous patients were compound heterozygous for missense mutations in *TELO2*, or had a combination of a missense and null allele (You et al. 2016), we interpreted the combination of a frameshift and a missense variant as likely disease‐causing. The missense c.2312T>C variant was ranked as probably damaging by all in silico prediction programs used (Polyphen: probably damaging, score = 1000; SIFT: damaging, score = 0; Mutation Taster: disease causing). Sanger sequencing confirmed that both variants segregated with the disease in the family: siblings were both compound heterozygous and the father and mother were heterozygous carriers of the variants in exon 14 and 20, respectively (Fig. 2A).

## Discussion

The salient clinical features of YHFS include: intellectual disability, microcephaly and short stature, global developmental delay with no regression of learned skills, dysmorphic facial features, abnormal movements and disturbed vision and hearing. The index patient presented herein may represent the severe end of the YHFS spectrum: in addition to the dysmorphic features, seizures and abnormal visual and auditory function which she shares with the original YHFS patients, she has severe growth retardation and disproportionate microcephaly of prenatal onset with postnatal persistence, reminiscent of the microcephalic primordial dwarfism syndromes. Her hands and feet are particularly small with tapering fingers, and overlapping toes with hypoplastic toenails. Moreover, she is profoundly globally developmentally delayed with no directed functions, unlike the original YHFS patients who at least developed some motor or speech abilities. Her phenotype is further characterized by additional congenital malformations of the renal system, an annular pancreas, and retinitis pigmentosa ‐ all previously unreported features. Her older sister had a cleft palate, like Patient II‐3 from Family 1 in the original report (You et al. 2016). She passed away too early to manifest further features aside from the typical microcephaly, growth retardation and dysmorphic facial features. We have shown that she carries the same *TELO2* mutations, although her presentation was not identical to her sister's. Intra‐familial variability in the clinical presentation was noted in the original Family 1 described by You et al. (2016).

The underlying molecular pathogenesis of YHFS is still not completely understood. Exactly how these mutations in *TELO2* influence the overall structure and function of the TELO2 protein and its interaction with its binding partner, remains to be elucidated. Moreover, how the resultant predicted destabilization of the TTT‐complex causes the YHFS phenotype, requires further investigation. Families 1, 2 and 3 in the original YHFS report carried compound heterozygous missense mutations, whereas the affected boy from Family 4 had three mutations, one missense mutation on one chromosome (the same as in Family 1) and a complex allele comprising a missense and a splice‐site mutation on the other chromosome, which was thought to be a null allele. His clinical picture, however, is also not as severe as that seen in our patient (You et al. 2016). Which mechanisms underlie severity and the intra‐familial variability warrants further investigation.

This case report serves to (i) add to the clinical delineation of the new YHFS syndrome to include features in keeping with a form of microcephalic primordial dwarfism on the severe end of the clinical spectrum, and to (ii) expand the mutational spectrum of *TELO2* to include two novel mutations. Further patients are needed to more accurately delineate the complete clinical spectrum of YHFS.

## Conflict of Interest

Authors declare no conflict of interest.

## Web Resources

1000Genomes: http://browser.1000genomes.org/index.html; Exome variant server: http://evs.gs.washington.edu/EVS/; Exome Aggregation Server (ExAC): http://exac.broadinstitute.org/; Exome Sequencing Project (ESP): https://esp.gs.washington.edu; Varbank: https://varbank.ccg.uni-koeln.de/
